# Expressions of the satellite repeat HSAT5 and transposable elements are implicated in disease progression and survival in glioma

**DOI:** 10.55730/1300-0152.2700

**Published:** 2024-07-01

**Authors:** Sıla Naz KÖSE, Tutku YARAŞ, Ahmet BURSALI, Yavuz OKTAY, Cihangir YANDIM, Gökhan KARAKÜLAH

**Affiliations:** 1 Department of Genetics and Bioengineering, Faculty of Engineering, İzmir University of Economics, İzmir, Turkiye; 2 İzmir Biomedicine and Genome Center (IBG), İzmir, Turkiye; 3 İzmir International Biomedicine and Genome Institute (IBG-İzmir), Dokuz Eylül University, İzmir, Turkiye

**Keywords:** Glioma, glioblastoma, transposon, satellite repeat, HSAT5, survival

## Abstract

The glioma genome encompasses a complex array of dysregulatory events, presenting a formidable challenge in managing this devastating disease. Despite the widespread distribution of repeat and transposable elements across the human genome, their involvement in glioma’s molecular pathology and patient survival remains largely unexplored. In this study, we aimed to characterize the links between the expressions of repeat/transposable elements with disease progression and survival in glioma patients. Hence, we analyzed the expression levels of satellite repeats and transposons along with genes in low-grade glioma (LGG) and high-grade glioma (HGG). Endogenous transposable elements LTR5 and HERV_a-int exhibited higher expression in HGG patients, along with immune response-related genes. Altogether, 16 transposable elements were associated with slower progression of disease in LGG patients. Conversely, 22 transposons and the HSAT5 satellite repeat were linked to a shorter event-free survival in HGG patients. Intriguingly, our weighted gene coexpression network analysis (WGCNA) disclosed that the HSAT5 satellite repeat resided in the same module network with genes implicated in chromosome segregation and nuclear division; potentially hinting at its contribution to disease pathogenesis. Collectively, we report for the first time that repeat and/or transposon expression could be related to disease progression and survival in glioma. The expressions of these elements seem to exert a protective effect during LGG-to-HGG progression, whereas they could have a detrimental impact once HGG is established. The results presented herein could serve as a foundation for further experimental work aimed at elucidating the molecular regulation of glioma genome.

## 1. Introduction

Gliomas are the most common type of central nervous system tumors representing approximately 80% of all malignant brain tumors (Ostrom et al., 2017). They can vary at genetic and epigenetic levels causing differences in tumor aggressiveness and hence a heterogeneous response to therapy (Noushmehr et al., 2010; Sturm et al., 2014; Sakthikumar et al., 2020; Garcia-Fabiani et al., 2021). Gliomas are histologically classified as astrocytic, oligodendroglial, or mixed (astrocytic and oligodendroglial) according to the cell type from which they originate (Louis et al., 2016). Importantly, they can be low-grade (LGG) or high-grade (HGG) according to their malignancy levels. LGGs typically arise de novo without any prior precursor or lower-grade lesions, and they have a better prognosis with approximately 75% five-year survival rates, while HGGs are grade III/IV gliomas generally differentiated from those of lower grades with a much lower five-year survival rate of only 5% (Stupp et al., 2009; Crespo et al., 2015; Teng et al., 2022). Even though gliomas could arise from different cell types as mentioned above, they exhibit similar molecular characteristics based on their grade (i.e. LGG or HGG) and mutational statuses (Arcella et al., 2020).

Numerous studies have attempted to unravel histological and molecular differences between LGG and HGG to facilitate the identification of potential biomarkers and therapeutic targets. LGGs are usually characterized by IDH, ATRX, and TP53 alterations, as well as CDKN2A hypomethylation and 1p/19q codeletion (Parsons et al., 2008; Brat et al., 2015; Louis et al., 2016), whilst progression to HGG can be characterized by PTEN mutations, EGFR amplifications and MGMT promoter hypermethylation (Wang et al., 2016; Brito et al., 2019). It is noteworthy that some of these alterations may have distinct and sometimes opposite prognostic implications in LGG and HGG. Several mutations were found to be associated with better outcomes in LGG whilst presenting worse outcomes in HGG (Eckel-Passow et al., 2015; Juratli et al., 2018). Moreover, dysregulations in signaling pathways, mutations in oncogenes and tumor suppressors, and impaired epigenetic mechanisms may lead to genomic instability and prompt the chromatin of a glioma cell for further malignant progression (Louis et al., 2016; Mackay et al., 2017). Pivotal changes in chromatin architecture were observed for various types of gliomas and they were related to tumor aggressiveness, as well as patient survival (Mack et al., 2019; Wang et al., 2021; Garrett et al., 2022; Yang et al., 2022). Further insights into the dynamics of these chromatin aberrations would therefore pave the way for designing more effective treatment strategies.

There are a multitude of layers that influence chromatin architecture, one of which certainly is the content of DNA. At least 50% of the human genome is made up of repeat elements (International Human Genome Sequencing Consortium, 2001) and their transcription levels reflect the status of chromatin (Probst et al., 2010; Jagannathan and Yamashita, 2017). Even though their exact contribution to subnuclear regulation is yet to be characterized, expression of repeats was generally linked to vital biological processes such as heat stress response (Porokhovnik et al., 2021) and cellular senescence (Karakülah and Yandım, 2020). More importantly, tightly regulated expressions of these elements were particularly implicated in embryonic development (Ulitsky et al., 2011; Yandım and Karakülah, 2019a). Whereas tandemly repeated arrays such as the satellites could affect kinetochore formation and hence chromosomal stability (Vos et al., 2006; Zhu et al., 2011), interspersed elements such as DNA transposons or retrotransposons (i.e. long interspersed nuclear elements [LINEs], short interspersed nuclear elements [SINEs], long terminal repeats [LTRs], and human endogenous retroviruses [HERVs]) may act as a mutagenic force (Lee et al., 2012; Helman et al., 2014). Therefore, not surprisingly, dysregulated transcription levels of both satellites and transposons were reported to be potentially disease-causing in various cancers including but not limited to breast cancer (Zhu et al., 2011; Yandım and Karakülah, 2019b), hepatocellular carcinoma (Karakülah and Yandım, 2021), and pancreatic cancer (Ting et al., 2011; Yandım and Karakülah, 2022).

Despite reported changes in global chromatin and genome regulation, there have only been limited investigations into the contribution of repeat elements to the molecular pathogenesis and prognosis of gliomas. Ohka et al. reported that hypomethylation events in LINE elements were associated with shorter overall survival in glioma patients (Ohka et al., 2011). In addition, it was indicated that transcriptions of Alu elements were posttranscriptionally edited in oligodendroglioma (Hwang et al., 2022). Another recent study focusing on endogenous retroviruses showed that 43 HERV elements were upregulated in HGG compared to healthy brain tissue with implicated effects on proximal gene regulation (Koso et al., 2012; Yuan et al., 2021). On the other hand, the specific types of repeats/transposable elements that could be associated with disease progression and survival in glioma have not yet been characterized. This could be partly because most of the published RNA sequencing (RNA-seq) data is prepared with a poly(A) bias in library preparation, which renders the analysis of repeat-arisen transcripts inconclusive (Solovyov et al., 2018).

To elucidate the particular links between repeat/transposable element expression and disease progression and survival in glioma patients, we employed a publicly available dataset (GSE184941) for which total RNA-seq had been performed without an mRNA bias on the tumor resection samples of 32 LGG and 34 HGG patients with IDH mutations (Supplementary file 1: Table S1) (Xiao et al., 2022; Khan et al., 2023). This was the only available relevant dataset that allowed the quantification of total transcripts arisen from repeat elements and transposons whilst also presenting patient survival attributes. We identified differentially expressed satellite repeats and transposons between LGG and HGG samples. We also performed survival analyses considering the expressions of all human RepBase repeats (Bao et al., 2015) with Cox-regression and log-rank tests (Bewick et al., 2004), revealing a subset of repeats that are potentially important for LGG to HGG progression and survival.

## 2. Results

### 2.1. Identification of differentially expressed genes and repeat elements in HGG in comparison to LGG

**Figure 1:**
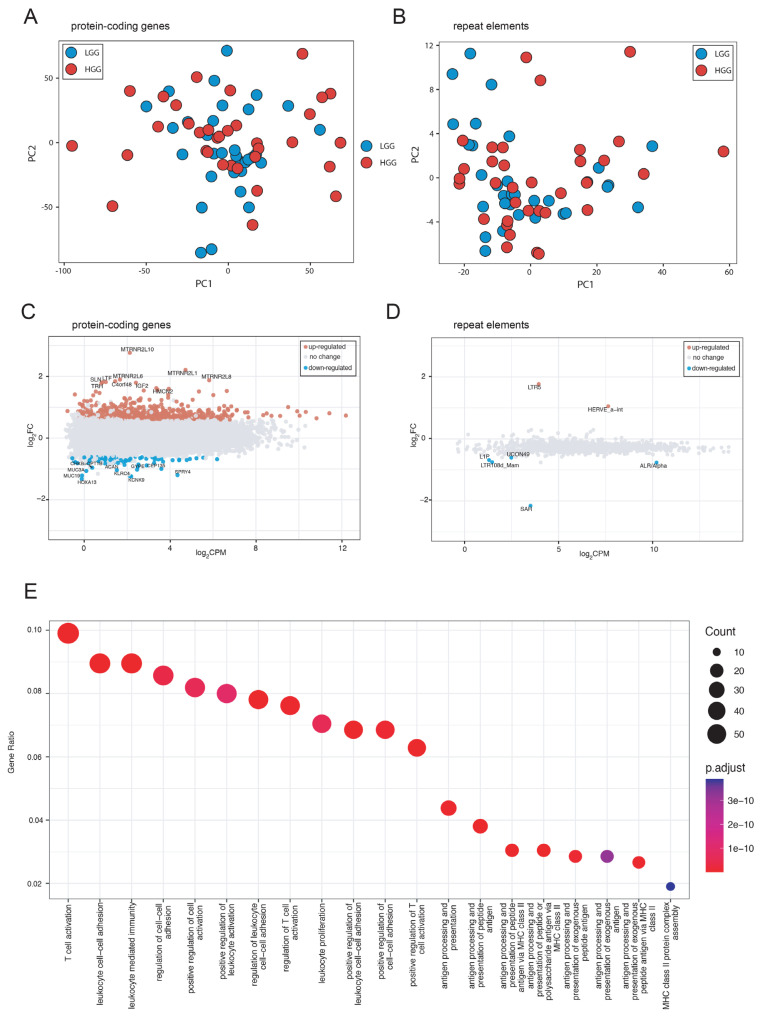
Comparison of gene and repeat expressions between low-grade (LGG) and high-grade (HGG) gliomas. **A** Principal component analysis (PCA) of protein-coding genes. **B** PCA of repeat elements **C** MA plot of protein coding gene expressions **D** MA plot of repeat element expressions. **E** Gene ontology analysis results on biological processes for genes differentially expressed in HGG relative to LGG.

To uncover the differences in gene and repeat/transposon expression, we quantified total RNA transcripts and performed a principal component analysis (PCA) (Figure 1 and Supplementary file 2: Figure S1). Even though a general distinction was observed in PCA analysis both for protein-coding gene expression and repeat arisen transcription levels between LGG and HGG samples (Figures 1A and 1B), the distribution of read percentages across patients were slightly different (Supplementary file 2: Figure S1A) with small changes in the abundance of repeat types giving rise to transcripts (Supplementary file 2: Figure S1B and S1C). When we applied a threshold of | log_2_(FC)| >0.6 | and false discovery rate (FDR) < 0.05, we identified 605 differentially expressed genes (Figure 1C and Supplementary file 3: Table S2) and 7 repeats (Figure 1D and Supplementary file 3: Table S3) in HGG samples when compared to LGG samples. A gene ontology analysis revealed that differentially expressed genes were mostly associated with biological processes implicating immunological response and cell-cell adhesion (Figure 1E, Supplementary File 4: Table S4), aligning well with previous literature (Xu et al., 2019). Retrotransposons LTR5 and HERVE_a-int were upregulated in HGG samples suggesting a possible role for them in the progression from LGG to HGG.

### 2.2. Identification of gene and repeat expressions implicated in disease progression and survival

**Figure 2:**
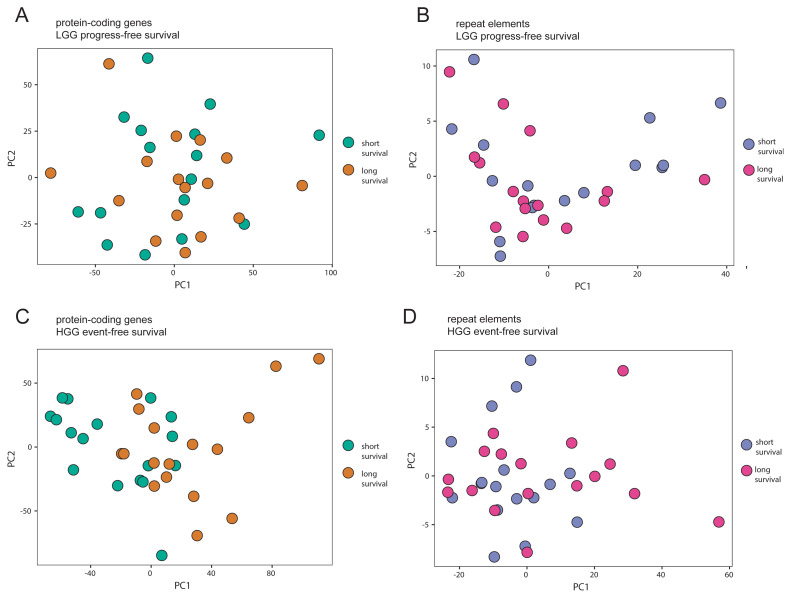
Principal component analyses (PCA) of the expressions of genes implicated in glioma progress and survival with a median cut-off. **A** PCA of samples associated with short and long progress-free survival in LGG considering the protein-coding gene expressions. **B** PCA of samples associated with short and long progress-free survival in LGG considering repeat element expressions. **C** PCA of samples associated with short and long event-free survival in HGG considering the protein-coding gene expressions. **D** PCA of samples associated with short and long progress-free survival in LGG considering repeat element expressions.

Gene expression signatures that influence survival times of patients provide invaluable information on the carcinogenesis and metastasis process in cancer (Sahu et al., 2021). Therefore, they have the potential to open new treatment avenues. To provide a detailed picture of the survival-related expression events where repeat-arisen transcripts were also considered in glioma, we performed a Cox regression analysis followed by a log-rank test (with a median progression/survival time cut-off) not only for the survival of HGG patients but also for the progression of LGG patients (to HGG). A PCA analysis showed a more pronounced separation between shorter and longer survival in the event-free survival analysis of HGG samples as opposed to progress-free survival analysis of LGG samples (Figure 2).

**Figure 3:**
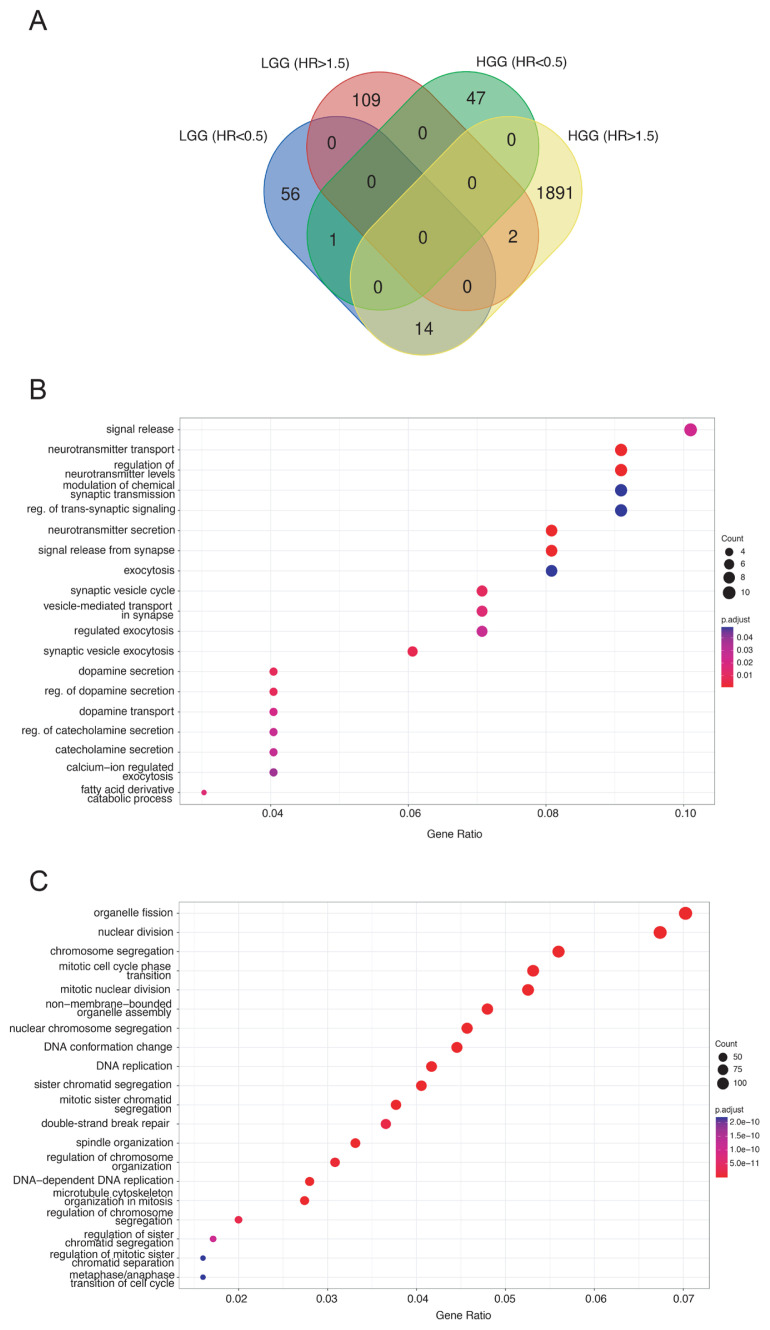
Progress-free survival genes in LGG and event-free survival genes in HGG. **A** Hazardous (HR>1.5, Log-rank p<0.05) and protective (HR<0.5, Log-rank p<0.05) genes based on Cox regression analyses and Log-rank test in LGG and HGG. **B** Gene ontology analysis of hazardous genes in LGG-to-HGG progression. **C** Gene ontology analysis of hazardous genes in HGG event-free survival.

Based on progress-free survival analysis in LGG samples, we identified 71 genes and 16 repeats (Cox Hazard Ratio [HR] < 0.5, Cox p-value < 0.05 and log-rank p-value < 0.05) that were associated with slower LGG to HGG progression (Table 1, Supplementary file 5: Tables S5 and S7). On the other hand, 111 genes, but none of the repeats, (Cox HR > 1.5, Cox p-value < 0.05 and log-rank p-value < 0.05) were found to be related to faster LGG to HGG progression (Table 1, Supplementary file 5: Tables S6 and S8). In the samples where progression to HGG took place, event-free (event: death) survival analysis revealed 48 genes, but no repeats (Cox HR <0.5, Cox p-value<0.05 and Log-rank p-value<0.05) were associated with longer survival (Table 1, Supplementary file 6: Tables S9 and S11). Interestingly, 1907 genes and 23 repeats (Cox HR >1.5, Cox p-value<0.05 and Log-rank p-value<0.05) were linked to shorter event-free survival in HGG (Table 1, Supplementary file 6: Tables S10 and S12). Only one gene (CASS4) was associated with longer progress-free survival in LGG, as well as longer event-free survival in HGG (Figure 3A). On the other hand, two genes (GPR156 and AGAP1), but no repeats, were associated with shorter survival both in progress-free analysis of LGG samples and event-free analysis of HGG samples at the same time. Intriguingly, 14 genes (IPO9, PLK1, DCAF4L1, ZNF345, LRRC75A, RAD18, BMP8A, LRR1, SETD6, AK2, MAP3K12, LMBR1L, ERCC6L, ZNF776) and one transposon (LTR18C) were related with longer time for LGG-to-HGG progression, yet in opposition, they were linked to shorter event-free survival in HGG samples. Gene ontology analyses revealed that LGG-to-HGG progression could be attributed to neurotransmitter release (Figure 3B, Supplementary file 7: Table S13), whereas survival in HGG could be related to various cell cycle events including spindle organization, chromosome segregation, and nuclear division (Figure 3C, Supplementary file 8: Table S14).

### 2.3. Weighted gene co-expression network analysis (WGCNA) reveals the expression of HSAT5 satellite as a potential key player in HGG

**Figure 4:**
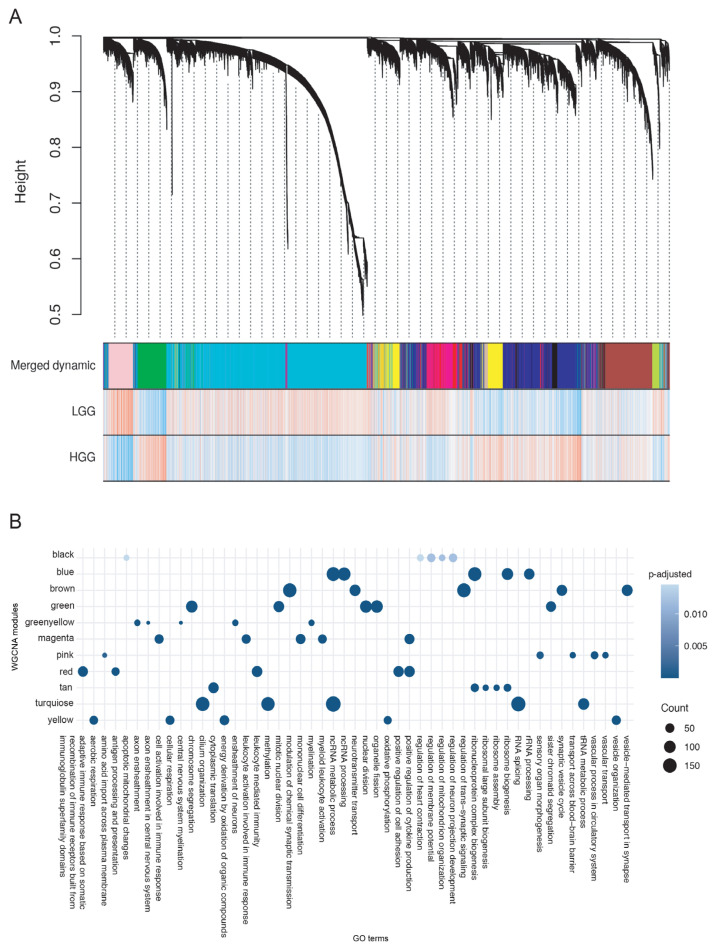
Weighted Gene Co-expression Network Analysis (WGCNA) of gene and repeat expressions. A WGCNA modules B Gene ontology analysis of the genes in WGCNA modules.

In order to further elucidate specific gene expression correlation patterns that could be distinctly related to LGG or HGG and to provide a better insight into the cooperation of repeat expression in these patterns, we performed weighted gene co-expression network analysis (WGCNA) (Zhang and Horvath, 2005; Cantone and Fisher, 2013). Our pipeline contained a pool approach where not only the transcript levels of genes but also those of repeats were analyzed altogether. Overall, 13 modules that contained genes and repeats were identified and these were represented with different colors (Figure 4A, Supplementary file 9: Table S15). Tan module was specifically emphasized in LGG samples, and the green module was particularly associated with HGG. Whereas the tan module contained no repeats but included 185 genes, the green module comprised only the satellite repeat HSAT5, no transposons, and 981 genes. The majority (987) of repeat elements/transposons fell into turquoise module, which was emphasized in LGG samples and where all 16 repeats linked to longer LGG progress-free survival (Table 1) were also present. The blue module, on the other hand, was pronounced in HGG and contained 5 repeats, among which only L1M2a was linked to event-free survival in HGG. Greenyellow module was slightly pointing to LGG, and it contained only one repeat.

When a gene ontology analysis was run for all of the WGCNA modules, genes related to ribosome biogenesis and protein translation seemed to be enriched in the tan module (Figure 4B, Supplementary file 10). Interestingly, the green module displayed an enrichment in genes that were linked to nuclear division, chromosome segregation, and double-strand break repair. Turquoise module, where the majority of repeats reside, was enriched with genes linked to cilium organization, methylation and ncRNA metabolic process, splicing and tRNA metabolic process. The blue module was as well in an enrichment with ncRNA processing and ribosome biogenesis genes. On the other hand, the greenyellow module was related to myelination and neuron ensheathment.

**Figure 5:**
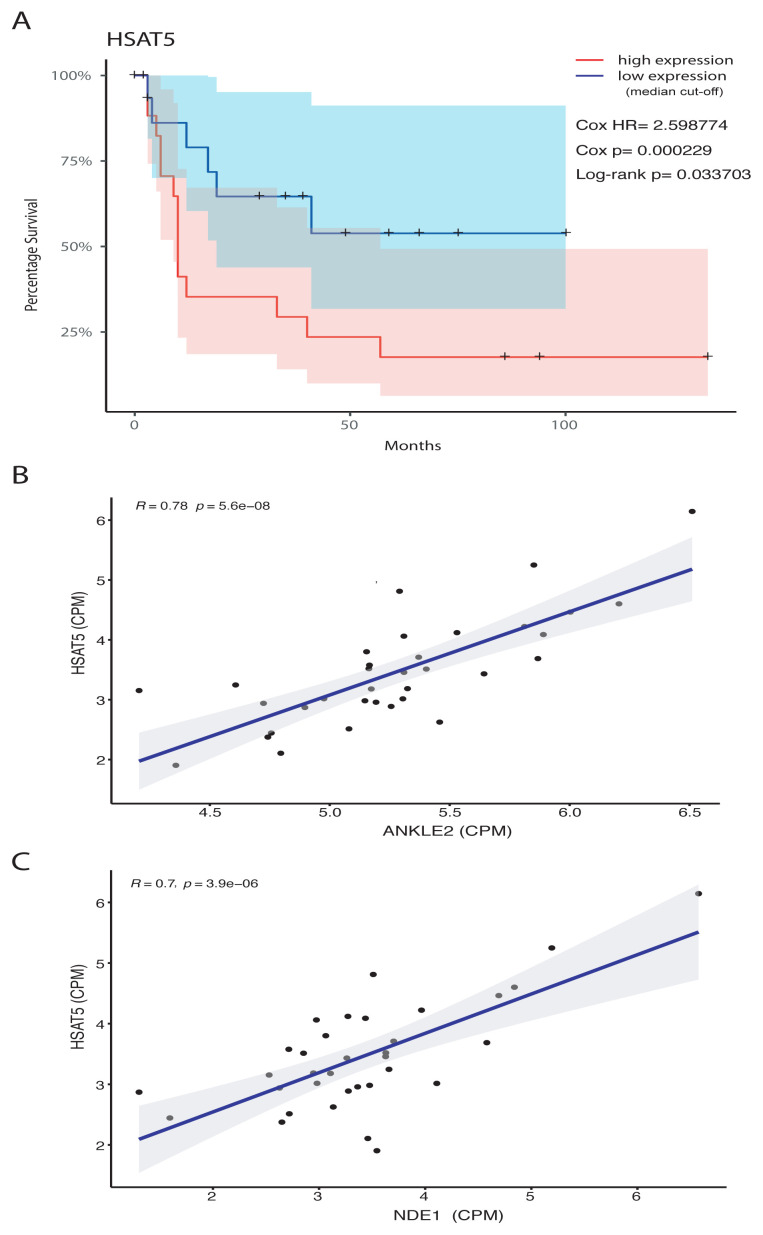
Survival analysis of HSAT5 expression and its correlation with key chromatin factors. A Kaplan-Meier event-free survival graph of HGG patients based on HSAT5 satellite repeat expression with a median cut-off. B,C Scatter plot representations showing the correlation of HSAT expression with the expressions of ANKLE2 and NDE1 genes in HGG patients.

Our interesting findings on HSAT5 satellite repeat which was linked to shorter event-free survival in HGG (Figure 5A) and the fact that it appeared in the same WGCNA module (green) with genes that were linked to similar biological phenomena (i.e. nuclear division and chromosome segregation) presented in Figure 3C for HGG event-free survival, prompted us to run a Pearson correlation analysis for the expression levels of HSAT5 and all protein-coding genes in the genome (Supplementary file 11). We found that 310 genes exhibited moderate-to-high level of correlation (r > 0.5) with HSAT5 expression. Strikingly, 195 (63%) of these 310 genes were among those significantly linked to shorter HGG event-free survival as presented in Table S10 (Supplementary file 6). A thorough literature search also showed that most of these genes were reported to be influential in glioma (Table 2). Some of these HSAT5-correlated genes including but not limited to ANKLE2 (Figure 5B) and NDE1 (Figure 5C) could be involved in the regulation of HSAT5 expression due to their crucial functions on chromatin (Chomiak et al., 2022; Sebastian et al., 2022).

## 3. Discussion

The complexity of molecular pathogenesis underlying gliomas makes them refractory to current treatment strategies. Notably, most of the patients that die due to gliomas suffer from HGG, which in most cases stem from a preceding LGG (Bogdańska et al., 2017). Therefore, understanding the particular events that cause progression to HGG from LGG is important in terms of projecting more insightful approaches toward gliomas. In this study, we uncovered not only differentially expressed protein-coding genes but also transcripts arisen from satellite repeats and transposons in HGG as opposed to LGG, shedding further light into disease progress. We found that genes related to immune response were differentially expressed in HGG as opposed to LGG, agreeing well with previous reports (Gabrusiewicz et al., 2016; Lu et al., 2019; Xiao et al., 2022; Khan et al., 2023). This could be a projected outcome due to immune cell infiltration as reported before (Domingues et al., 2016; Lin et al., 2021). Another interesting finding was the upregulated expression (in HGG) of HERV_a-int and LTR5 transposon, the latter of which was previously linked to cancer (Lee et al., 2012) and human preimplantation development (Yandım and Karakülah, 2019a).

Our progress-free survival analysis of LGG samples on gene expressions revealed that genes related to the regulation of neurotransmitter release, dopamine secretion and exocytosis were linked to faster progression to HGG. Even though genes within this context were not reported in some of the studies that analyzed clinical samples (Wang et al., 2020; Zhang et al., 2020), in vitro and animal studies previously suggested dopamine release as a potential factor in glioma (Li et al., 2014); however, its effect was not entirely understood (Lan et al., 2017). Another interesting finding in our study was that all repeat elements associated with LGG-to-HGG progression were those that had a hazard ratio of less than 0.5. In other words, transposon expression could potentially slow down the progression to HGG. This could be due to increased antigenicity and better immune response caused by transposon expression as suggested earlier (Kong et al., 2019; Yuan et al., 2021). In opposition, event-free survival analysis in HGG revealed that none of the repeat elements were associated with longer survival. However, it identified one satellite (i.e. HSAT5) and 23 transposons linked to shorter survival. Though this result suggests that repeat element expression may lead to opposite consequences in LGG and HGG, only one repeat (i.e. LTR18C) appeared in the survival analyses of both LGG (HR<0.5) and HGG (HR>1.5) groups. In line with this, the genes linked to shorter survival in HGG were also pointing out a different context as opposed to those in LGG. Genes with HR>1.5 were mostly linked to chromosome segregation and nuclear division, generally agreeing well with previous literature (Bredel et al., 2005; Phillips et al., 2006; Murat et al., 2008; Q. Wang et al., 2017).

In an effort to dissect the impact of repeat element expression on the molecular pathogenesis and survival in glioma, we not only presented LGG progress-free survival related repeats and HGG event-free survival linked repeats but also differentially expressed repeats in HGG samples in comparison to LGG. Yuan et al. (2021) reported differentially expressed repeats in tissue samples of HGG (GBM) patients as opposed to normal brain samples; however, the data they employed was prepared with an mRNA bias only detecting poly(A)+ transcripts. Our study utilizes sequencing data obtained from total RNA samples without the poly (A) bias (Khan et al., 2023) as previously recommended (Solovyov et al., 2018). Notably, LTR5 was reported to be upregulated both in a previous study (Yuan et al., 2021), which compares the HGG tissue with normal tissue, and in our study that compares with the HGG tissue with LGG, even though no survival related effect was detected. Another element we detected in HGG event-free survival analysis was HERVK3-int, which was reported to be upregulated in HGG samples with a potential effect on disease progression (Shah et al., 2022). Our holistic approach identified many other repeats/transposons previously unmentioned in the glioma literature. Perhaps, the most interesting one among these is the satellite repeat HSAT5, which predominantly resides in pericentromeric regions of chromosomes (Hillier et al., 2005; Hubley et al., 2016) and was also reported to be upregulated in cellular senescence (Karakülah and Yandım, 2020). Though no studies so far reported HSAT5 within the context of cancer, other satellites such as HSATI (Zhu et al., 2011), HSATII (Ting et al., 2011), and GSATII (Karakülah and Yandım, 2021) were linked to the molecular pathologies of solid cancers. Satellite repeats are known to play pivotal roles in maintaining heterochromatin architecture and kinetochore formation (Rošić et al., 2014; Nishibuchi and Déjardin, 2017) and their abnormal expressions were linked to genomic catastrophes in cell division (Zhu et al., 2011; Bersani et al., 2015; Kishikawa et al., 2016). Their usage as biomarkers is also being valuably considered (Özgür et al., 2021). Our results point out HSAT5 satellite repeat as a potential prognostic biomarker in HGG survival. More importantly, its cooperative expression levels with HGG-specific genes that are known to involve in chromosome segregation and nuclear division could implicate its potential in the molecular pathology of this devastating disease. The relationship of HSAT5 DNA or RNA with chromatin-related factors such as NDE1 and ANKLE2 could illuminate the mechanism of HSAT5 expression further.

## 4. Conclusion

In this study, we performed a comprehensive analysis on the expressions of repeat elements covering both satellite repeats and transposons to figure out those implicated in glioma progression and survival. Our results interestingly revealed that the expressions of transposable elements could be linked to slower LGG to HGG progression, yet this beneficial effect is overturned once the progress to HGG is manifested. One explanation for the beneficial effect in LGG could be the improved antigenicity caused by repeat elements, which provides better immune response. On the other hand, once the progression to HGG takes place, repeats could be contributing to dysregulated events in chromosome segregation and cell cycle as mostly the results on HSAT5 satellite repeat here suggested. Results presented here could serve as a guide for further experimental work in understanding the molecular basis of gliomas further.

## 5. Methods

### 5.1. Data acquisition and preprocessing

We obtained raw sequencing data from the Sequence Read Archive (SRA) database (Leinonen et al., 2011) for 66 glioblastoma patients (SRA accession: SRP339173; GEO accession: GSE184941) using the “fastq-dump -gzip -skip-technical -readids -dumpbase -clip -split-3” command of the SRA Tool Kit v.2.9.0. We collected the GRCh38 human genome assembly and comprehensive gene annotation (release 34) in gene transfer format from the GENCODE^1^ website (Frankish et al., 2021). We obtained genomic coordinates of repeat instances across the genome from the RepeatMasker^2^ website. We aligned paired-end reads from each tumor sample to the human reference genome using the Rsubread package v1.34.7 (Liao et al., 2014) in the R statistical computation environment with default parameters. We used SAMtools suite v1.3.1 (Li et al., 2009) to sort and index the binary alignment map files. We counted reads using the featureCounts function of the Rsubread package (Liao et al., 2014), which allowed us to differentiate between exonic and repetitive regions. We merged repeat and gene counts into a single expression matrix, and calculated counts per million (CPM) values for each gene and repeat element across all tumor samples. We excluded any gene or repeat element that was not expressed above 1 CPM in at least 25% of the tumor samples from further analysis. Besides, when filtering for HGG and LGG samples, IDH mutants and percentage of total mapped reads greater than 70 were included in both samples. To improve the accuracy of estimated repeat element expressions, we only considered sequencing reads that overlapped nonexonic regions and uniquely mapped to DNA, LINE, SINE, LTR, and satellite repeat regions.

### 5.2. Coexpression network analysis of protein-coding genes and repeat elements

To perform coexpression network analysis, we used the R package WGCNA v1.47 (Langfelder and Horvath, 2008). Adjacencies between all protein-coding genes and repeats across samples were calculated. In this step, CPM values of genomic features were used as input. We designated the soft threshold value as 12 and the average linkage hierarchical clustering method was selected for grouping the genomic features with similar expression patterns. Additionally, the dynamic tree cut algorithm (Langfelder et al., 2008) was utilized to determine network modules and minimum network module size was set to 30. Gene ontology (GO) enrichment analysis of each network module was performed with the R package clusterProfiler v3.18.0 (Yu et al., 2012).

### 5.3. Statistical analysis and graphical representation

Statistical analysis and graphical representation were performed using the R environment^3^ for the LGG and HGG samples. Differential expression (DE) analysis of genomic features was performed using the edgeR package (version 3.28.2) (Robinson et al., 2010). We obtained a PCA plot of samples to visualize clusters of LGG and HGG samples based on their similarities (quantile median = 0.50). We used the log rank test (logrank_test function of the coin package, version 1.4.1) (Royston et al., 2019) to test the statistical significance of survival time differences between groups and the Cox regression analysis (coxph function of the survival package, version 3.2.11) to estimate hazard ratios (HR). The Kaplan-Meier curves were drawn using the survfit function to show the probabilities of survival for a certain time interval. We used the ggplot2 package (version 3.3.6) to create other graphical representations.

## Supplementary Data

























## Figures and Tables

**Table 1:** Significant repeat elements linked to patient survival in LGG and HGG.

Low-grade glioma (LGG)						High-grade glioma (HGG)					
Longer progress-free survival (Log-rank p<0.05)	**Repeat**	**Cox HR (<0.5)**	Shorter progress-free survival (Log-rank p < 0.05)	**Repeat**	**Cox HR (>1.5)**	Longer event-free survival (Log-rank p < 0.05)	**Repeat**	**Cox HR (<0.5)**	Shorter event-free survival (Log-rank p < 0.05)	**Repeat**	**Cox HR (>1.5)**
	LTR1F2	0.226566207		None			None			hAT-16_Crp	3.841230728
	MER107	0.246705973								HERV1_LTRb	3.772926264
	MER1B	0.253616041								MLT1E3-int	3.693884586
	LTR57	0.342074886								L1M2a	3.661849157
	LTR1C	0.362824242								L1M3f	3.640586269
	MER130	0.379093156								CR1-11_Crp	3.092362862
	LTR40c	0.388071793								MER95	2.958200736
	LTR1	0.394057275								UCON97	2.91316852
	LTR1A2	0.394722746								MSTC-int	2.886032148
	LTR2752	0.398195734								MER70-int	2.688166503
	LTR18C	0.421946618								HERVI-int	2.664554289
	LTR27C	0.449130349								L1MDa	2.613437037
	LTR35A	0.458301493								HSAT5	2.598739703
	MER9B	0.465923328								LTR75	2.456202539
	LTR27	0.476049565								HERVK3-int	2.414286886
	LTR7C	0.491857327								L1MB2	2.396560074
										L1M8	2.340619317
										LTR70	2.3387068
										L1M4a1	2.31165162
										LTR18C	2.18835578
										MER51-int	2.165050709
										HERVKC4-int	1.908960599
										Kanga1c	1.852908386

**Table 2:** Genes, whose expressions significantly correlated with that of HSAT5 satellite repeat, and their known associations with gliomas. Gene names written in bold characters indicate a direct link with survival in HGG as represented in Figures 1A and 1C.

Gene name	p-values	Pearson’s corr. coef. (r)	Direct association with glioma
**ANKRD20A1**	6.48E+02	0.723955	Membrane shaping (Wolf et al., 2019)
**QTRT2**	2.01E+02	0.712178	Ferroptosis (Sallam et al., 2022)
**ANKRD20A3P**	1.72E+04	0.688139	-
**IGF2BP2**	2.47E+04	0.683868	Chemoresistance (Han et al., 2022)
**RALY**	2.09E+04	0.656957	-
**CCDC150**	3.09E+05	0.651718	-
TRIO	3.79E+05	0.648938	Cell migration, invasion and disease outcome (Salhia et al., 2008)
ODF2	4.04E+04	0.648070	Hypoxia (Baghbani et al., 2013)
**ANKLE2**	6.30E+05	0.641924	Apoptosis and survival (Wang et al., 2018)
**SHC4**	7.97E+05	0.638604	EMT, disease progression and outcome (Tilak et al., 2021)
**TTF2**	1.27E+06	0.631903	Cell cycle and repair mechanisms, extracellular matrix organization (Chai et al., 2019)
**ANKRD11**	1.53E+06	0.629146	-
**GSDMB**	1.88E+06	0.626186	Immune cell infiltration (Lin J et al, 2022)
**MCM8**	1.91E+06	0.625905	Stemness (Wang et al., 2021)
**SGCA**	2.22E+06	0.623704	ECM signaling (Balvers et al., 2013)
**NDE1**	2.28E+06	0.623247	Cell proliferation, migration, and disease prognosis (Chen et al., 2017; Song et al., 2019)
**DIAPH3**	2.94E+06	0.619450	Chemoresistance (Chehade et al. 2022)
**PIAS2**	3.46E+06	0.616934	Posttranslational modification (Li and Meng, 2021)
**PLCG1**	3.74E+06	0.615770	Disease prognosis (Li et al., 2022)
FUS	3.76E+06	0.615690	Angiogenesis (He et al., 2019)
**QRICH2**	4.18E+06	0.614060	-
**CAPRIN2**	4.62E+06	0.612492	-
**WDR62**	4.98E+05	0.611313	-
**SFI1**	5.52E+06	0.609695	-
**GNL3L**	6.79E+06	0.606424	Survival (Sun et al., 2021)
**POLE**	7.04E+06	0.605863	Genomic stability and disease progression (Erson-Omay et al., 2015)
**ZBTB49**	7.97E+06	0.603872	-
**ANAPC7**	8.56E+06	0.602737	Disease progression (de Groot et al., 2011)
PSMC3IP	9.16E+06	0.601632	Angiogenesis (Zhang et al., 2017)

## Data Availability

The dataset analyzed during the current study is available in the GEO repository, with accession number GSE184941.

## Footnotes

1
https://www.gencodegenes.org

2
http://www.repeatmasker.org/

3 v3.6.0; https://www.r-project.org/
