# Supplementary material for: Expressions of the satellite repeat HSAT5 and transposable elements are implicated in disease progression and survival in glioma
Source: Turk J Biol. 2024 Jul 1;48(4):242–56. doi: 10.55730/1300-0152.2700 (PMC11407350; doi:10.55730/1300-0152.2700)
Supplement: Supplementary file 2 [file Supplementary_File_2.pdf]

Supplementary Figure

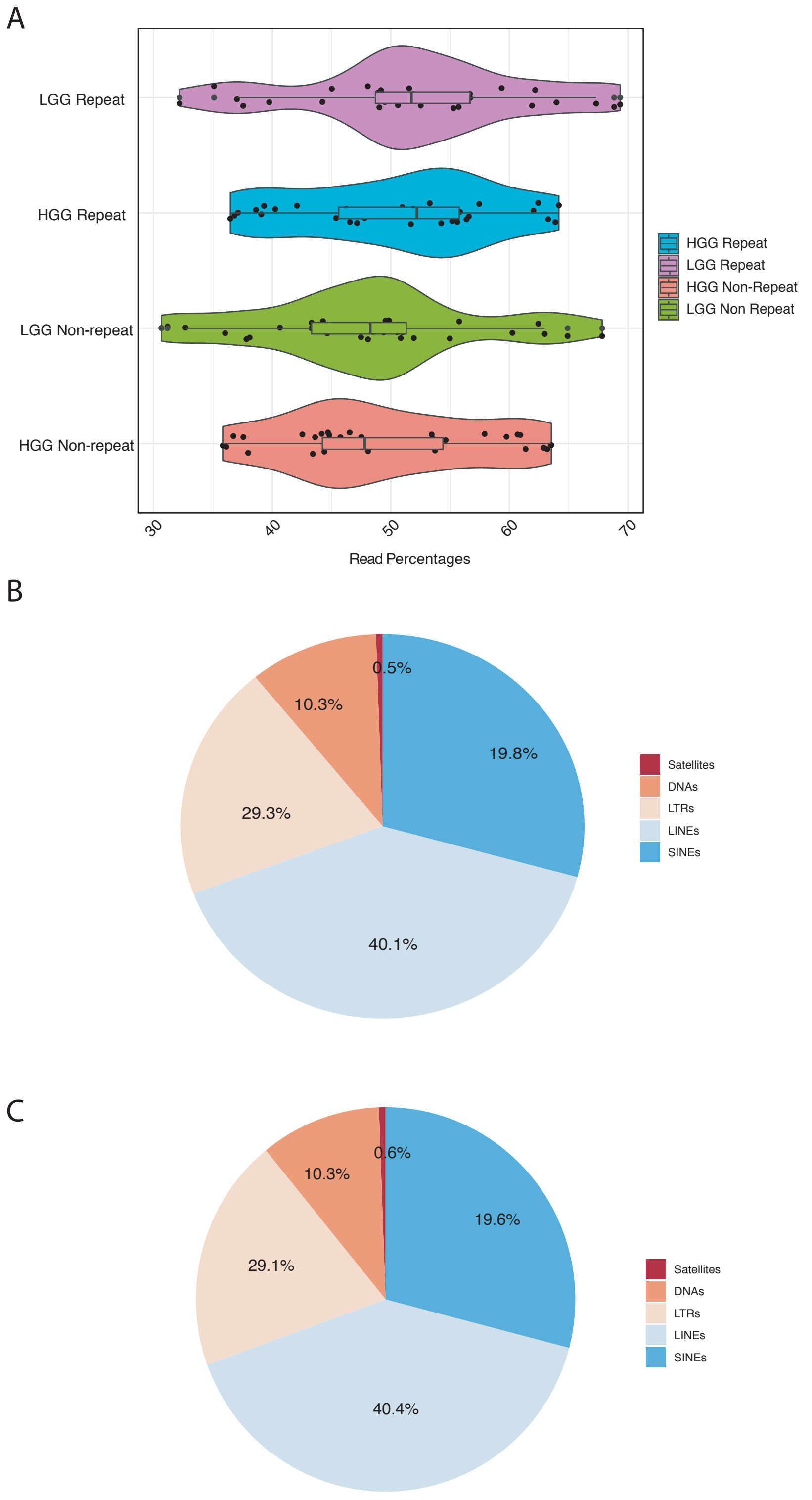

Figure S1. Distrubiton of read percentages of LGG and HGG samples. **A** Violin plot representing the distribution of read percentages of protein-coding genes and repeat elements in LGG and HGG samples. **B** Pie chart representing the percentages of repeat classes in LGG samples. **C** Pie chart representing the percentages of repeat classes in HGG samples.
