# Supplementary material for: Expressions of the satellite repeat HSAT5 and transposable elements are implicated in disease progression and survival in glioma
Source: Turk J Biol. 2024 Jul 1;48(4):242–56. doi: 10.55730/1300-0152.2700 (PMC11407350; doi:10.55730/1300-0152.2700)
Supplement: Supplementary file 12 [file Supplementary_Files_List.pdf]

Expressions of the satellite repeat HSAT5 and transposable elements are implicated in disease progression and survival in glioma

SUPPLEMENTARY FILES LIST

Supplementary File 1:

Table S1. SRR numbers of data used for the study.

Supplementary File 2:

Figure S1. Distrubiton of read percentages of LGG and HGG samples. **A** Violin plot representing the distribution of read percentages of protein-coding genes and repeat elements in LGG and HGG samples. **B** Pie chart representing the percentages of repeat classes in LGG samples. **C** Pie chart representing the percentages of repeat classes in HGG samples.

Supplementary File 3:

Table S2. Differentially expressed genes in HGG samples in comparison to LGG samples.

Table S3. Differentially expressed genes in HGG samples in comparison to LGG samples.

Supplementary File 4:

Table S4. Gene ontology analysis result list on biological processes of differentially expressed genes in HGG relative to LGG.

Supplementary File 5:

Table S5. Protective genes (HR<0.5) obtained in LGG progress-free survival analysis.

Table S6. Hazardous genes (HR>1.5) obtained in LGG progress-free survival analysis.

Table S7. Protective repeats (HR<0.5) obtained in LGG progress-free survival analysis.

Table S8. Hazardous repeats (HR>1.5) obtained in LGG progress-free survival analysis.

Supplementary File 6:

Table S9. Protective genes (HR<0.5) obtained in HGG event-free survival analysis.

Table S10. Hazardous genes (HR>1.5) obtained in HGG event-free survival analysis.

Table S11. Protective repeats (HR<0.5) obtained in HGG event-free survival analysis.

Table S12. Hazardous repeats (HR>1.5) obtained in HGG event-free survival analysis.

Supplementary File 7:

Table S13. Gene ontology analysis result list on biological processes of hazardous genes identified in LGG progress-free analysis.

Supplementary File 8:

Table S14. Gene ontology analysis result list on biological processes of hazardous genes identified in HGG event-free analysis.

Supplementary File 9:

Table S15. Genes listed in WGCNA modules.

Supplementary 10: Gene ontology analysis of genes identified in WGCNA modules pertaining to biological processes.

Supplementary File 11:

Table S16. Correlations of the expressions of protein-coding genes with HSAT5 satellite repeat expression.
